# Co-Benefits of Sustainable Forest Management in Biodiversity Conservation and Carbon Sequestration

**DOI:** 10.1371/journal.pone.0008267

**Published:** 2009-12-11

**Authors:** Nobuo Imai, Hiromitsu Samejima, Andreas Langner, Robert C. Ong, Satoshi Kita, Jupiri Titin, Arthur Y. C. Chung, Peter Lagan, Ying Fah Lee, Kanehiro Kitayama

**Affiliations:** 1 Graduates School of Agriculture, Kyoto University, Sakyo-ku, Kyoto, Japan; 2 Forest Research Centre, Sabah Forestry Department, Sandakan, Sabah, Malaysia; 3 Deramakot Forest Office, Sabah Forestry Department, Sandakan, Sabah, Malaysia; University of Zurich, Switzerland

## Abstract

**Background:**

Sustainable forest management (SFM), which has been recently introduced to tropical natural production forests, is beneficial in maintaining timber resources, but information about the co-benefits for biodiversity conservation and carbon sequestration is currently lacking.

**Methodology/Principal Findings:**

We estimated the diversity of medium to large-bodied forest-dwelling vertebrates using a heat-sensor camera trapping system and the amount of above-ground, fine-roots, and soil organic carbon by a combination of ground surveys and aerial-imagery interpretations. This research was undertaken both in SFM applied as well as conventionally logged production forests in Sabah, Malaysian Borneo. Our carbon estimation revealed that the application of SFM resulted in a net gain of 54 Mg C ha^-1^ on a landscape scale. Overall vertebrate diversity was greater in the SFM applied forest than in the conventionally logged forest. Specifically, several vertebrate species (6 out of recorded 36 species) showed higher frequency in the SFM applied forest than in the conventionally logged forest.

**Conclusions/Significance:**

The application of SFM to degraded natural production forests could result in greater diversity and abundance of vertebrate species as well as increasing carbon storage in the tropical rain forest ecosystems.

## Introduction

Selective logging of marketable, large trees has been a major mode of commercial timber production in Southeast Asian tropical rain forests. Tropical rain forests, designated to permanently produce timber with the application of such selective logging, are called “production forests” and occupy a large chunk of the tropical landscapes [1]. In case of Borneo, production forests cover approximately 35,000,000 ha, nearly one half of the total land area (K. Kitayama unpublished). Logging intensity by selective logging encompasses the amount of both harvested timber as well as collateral mechanical damages to residual stands. Comprehensive approaches to reduce harvesting intensity of selective logging (both in terms of harvested amount and collateral damages) determine the fate of the dynamics of production forests on a regional scale.

Historically, the principle of sustainability dictates that a production forest should be managed to limit as much undesirable damage as possible to the residual stand and overall ecosystem, and the detailed regulations (such as annual allowable cut, improving logging methods, forest zoning) were determined based on the concepts of guiding principles [2]. For example, the annual allowable cut has been determined to regulate the yield and rotation period in relation to expected regrowth based on ecological and silvicultural information and to maintain timber resources at a sustainable level [2], [3]. However, such regulations could not provide loggers with enough incentives to protect their timber resources and loggers harvested, in a short time, a much greater amount of timber than specified by the regulations. This also caused disproportionately greater collateral damages to the residual stands [4]–[6]. Consequently, extensive areas of highly degraded tropical rain forests cover large areas of tropical landscapes of Borneo and elsewhere in tropical Asia [1].

Recently, sustainable forest management (SFM) combined with reduced-impact logging (RIL) and forest certification has been applied in some production forests in Sabah, Malaysian Borneo [7], [8]. RIL consists of careful pre-harvest planning and improved harvesting techniques [9], but also involves reduced harvest and post-harvest silvicultural treatments [7], [8]. The higher costs and the reduced timber yields can be compensated by the economic benefits of forest certification, which result in an improved market access and in an increased unit log price [10]. Quantitative criteria and standards used in the auditing of a certification process bring loggers to comply with the regulations. Investigations have revealed that such SFM is beneficial not only in maintaining timber resources but also in conserving the carbon stock in the residual stands [4]–[6], [11]. In this paper, we demonstrate that SFM results in greater diversity and higher densities of some forest-dwelling vertebrate species while also increasing ecosystem carbon storage relative to forests, in which unregulated (conventional) logging is applied. SFM is a broader management concept which combines RIL techniques with a longer cutting cycle in the entire management unit. We therefore examine the effects of RIL as well as the longer cutting cycle on a landscape level as a net effect of SFM.

### Study Sites and Contrasting Logging Histories

Study sites are the production forests of the Deramakot and Tangkulap Forest Reserves in Sabah, Malaysian Borneo (5°14–30′N, 117°11–36′E). In this paper we use the term “reserve” for land designated for production. Deramakot (551 km^2^) and Tangkulap (275 km^2^) are located adjacent to each other and are covered by lowland mixed dipterocarp tropical rain forest. Deramakot is a sustainably managed forest with reduced impact logging, while Tangkulap is a forest damaged by conventional logging at the time of our investigation (2001–2007). We demonstrate the effects of SFM (especially RIL) on carbon density in terms of the sum of above-ground, fine roots, and soil organic carbon and the diversity of forest-dwelling medium to large-bodied vertebrate species at a landscape-scale based on a comparison between the two reserves.

Deramakot and Tangkulap were originally licensed for logging starting in 1956 and 1970s, respectively, and the conventional logging commenced there [12]. In 1989, Deramakot was chosen by the Sabah State Government as a model site to develop a sustainable forest management system and all logging activities were suspended thereafter. A new management system with reduced-impact logging was implemented in 1995. Deramakot was certified by Forest Stewardship Council in 1997 for its well management.

Deramakot is now divided into 135 compartments of varying size (approx. 500 ha each), and annual harvests are planned on a compartment basis [8]. 17 of these compartments (3,473 ha in area) are reserved for conservation (not to produce logs) [12]. About two to four compartments are harvested annually using RIL methods with a planned rotation period of 40 years. Average annual yield in Deramakot during 1995–2004 was about 23 m^3^ ha^−1^ for the net harvested area of two to four compartments. Based on the available data, the average timber production in Deramakot was much higher with 109 m^3^ ha^−1^ during the pre-RIL era in 1959–1968 [12].

By contrast, the Tangkulap forest reserve was repeatedly logged using a conventional logging technique until 2001, when the government suspended all logging activities. As there are no reliable statistics for the log production in Tangkulap, we reconstructed the logging history of Tangkulap in comparison to Deramakot, which served as a reference, using Landsat satellite data.

**Figure 1:**
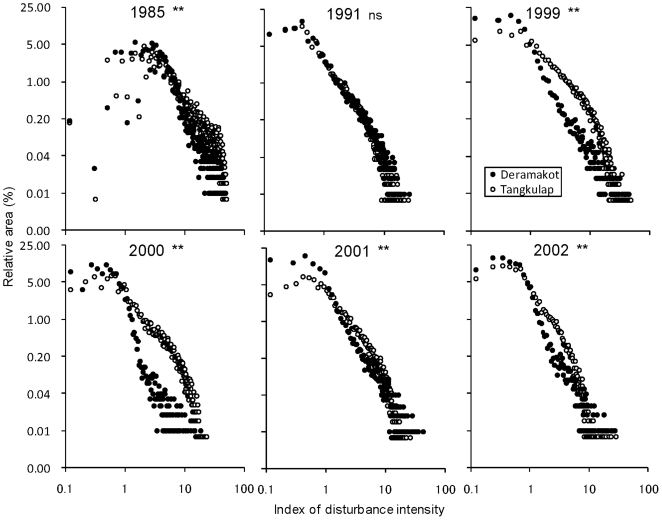
Disturbance history of the two forest reserves between 1985 and 2002. Area - disturbance intensity curves of Deramakot and Tangkulap from 1985 until 2002, based on the slope values of the red band reflectances of the Landsat MSS, TM and ETM+ imagery data. The percent relative area (*y*-axis) is plotted against the disturbance intensity (*x*-axis) double logarithmically. The shape of the curves indicates the impact of disturbance, with convex curves indicating higher disturbance impact than concave curves. Comparison of shape of the curves between the two reserves: **, p<0.01; ns, not significant.

To demonstrate the logging history in terms of forest management system in the two forest reserves, area-disturbance intensity curves were compared across six periods during 1985–2002 in the two reserves (Figure 1). Disturbance intensity is estimated based on the amount of bare soil (opened crown cover) in contrast to vegetation (see Methods). In Deramakot, the volume of harvested log was consistently lower than 15,000 m^3^ yr^−1^ with a net harvested area of less than four compartments after 1995 [8]. The area-disturbance intensity curves of Deramakot were indeed mutually similar across the four consecutive years from 1999 until 2002 (Figure 1), indicating that the curves could represent logging intensity and area. The curves were generally more convex in Tangkulap than in Deramakot (P<0.01) except for 1991, suggesting that much heavier logging occurred in greater areas in Tangkulap at these periods due to unregulated conventional logging. The curve in Tangkulap became steeper reflecting the governmental suspension of logging activities after 2001. In 1991 when logging was being suspended in Deramakot, the curves of the two reserves did not differ from each other (P > 0.01), suggesting that the logging intensity and areas in Tangkulap were also restricted.

Carbon density in the forests was estimated for the year 2001 based on aerial-imagery interpretations and ground-based measurement. As has been demonstrated in Figure 1, logging intensity was most contrasting between the two reserves probably after 1995 until 2001. Accordingly, the difference in carbon stock between the two reserves represents a net effect of the application of SFM with RIL. Vertebrate species were investigated in 2008, i.e. seven years after the suspension of logging in Tangkulap. Therefore, any positive effects of RIL on vertebrate species can be a conservative estimate.

## Results and Discussion

### Carbon Density

**Figure 2:**
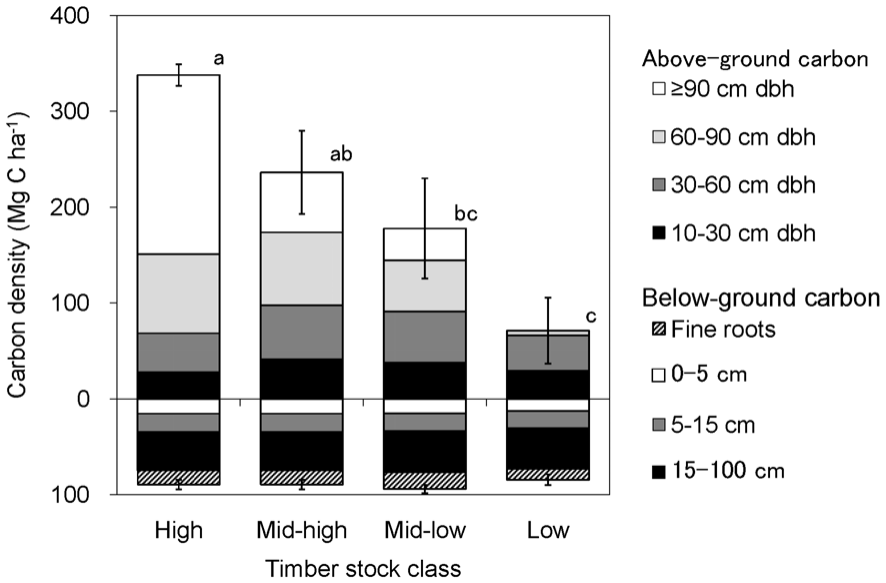
Carbon density in four timber stock classes. Carbon density is the sum of above-ground, fine roots, and soil organic carbon. Above-ground carbon density was shown by four dbh (diameter at breast height) classes (i.e., 10–30, 30–60, 60–90, and ≥90 cm dbh), and soil organic carbon density was shown by three soil depth classes (i.e., 0–5 cm (O and A horizons), 5–15 cm (AB horizon), and 15–100 cm deep (B horizon)). Timber stock class sharing the same letters did not significantly differ in carbon density at p<0.05.

Carbon density in terms of the sum of above-ground, fine roots, and soil organic carbon varied greatly among forest stands reflecting the past logging intensity and recovery processes (Figure 2). Carbon density varied from 156±36 Mg C ha^−1^ in a low-stocked forest to 427±11 Mg C ha^−1^ in a high-stocked forest, corresponding to a highly degraded forest harvested by conventional logging and to a pristine forest, respectively.

**Figure 3:**
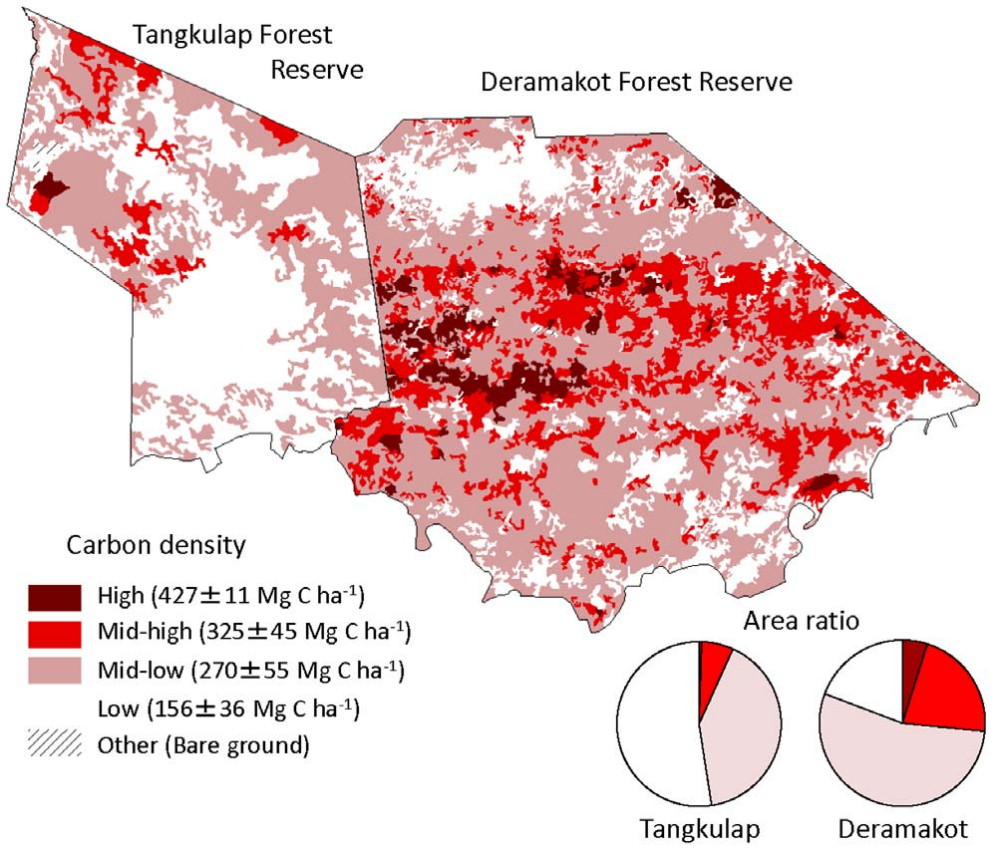
Benefit of sustainable forest management on carbon sequestration at a landscape level. Map of Deramakot (right side) and Tangkulap Forest Reserve (left side), showing the distribution of forest stands with four timber stock classes. Lower circular graphs indicate the proportion of land area of each timber stock class by the reserve.

Figure 3 demonstrates the distribution of forest stands with four timber stock classes based on aerial-imagery interpretations as of 2001. The two areas consist of complex mosaics, reflecting past logging activities as well as vigorous post-logging regrowth. High and mid-high stock classes together covered 27% of the area in Deramakot, but only 7% in Tangkulap (Figure 3). Low stock class covered 19% in Deramakot, while 52% in Tangkulap. Approximately one half of the area was covered by the mid-low stock class in both areas (54% in Deramakot and 41% in Tangkulap). In Deramakot, the conservation area covered 8.9%, 7.0%, 6.6%, and 5.8% of the land area of high, mid-high, mid-low, and low stock classes, respectively.

Estimated mean carbon density in above-ground vegetation was 178±15 and 126±17 Mg C ha^−1^ in Deramakot and Tangkulap, respectively. The difference of 52 Mg C ha^−1^ can be considered a net effect of SFM on a landscape level, reflecting the reduced harvest intensities, reduced logged area, and vigorous post-logging regrowth in Deramakot. In contrast, there was little difference in below-ground carbon density in the two forests (91±1.0 Mg C ha^−1^ in Deramakot and 89±0.8 Mg C ha^−1^ in Tangkulap). The application of SFM has resulted in a net gain of 54 Mg C ha^−1^ at a landscape scale during 1989–2001 (between the time when SFM commenced in Deramakot and the time when aerial photographs were taken for carbon estimation).

### Wildlife Diversity

We investigated the frequency and diversity of medium to large-bodied (>1 kg weight) vertebrates throughout Deramakot and Tangkulap using automatic heat-sensor cameras. We systematically chose 29 plots at 5 km intervals throughout the two forest reserves (20 and 9 plots in Deramakot and Tangkulap, respectively), and three cameras were randomly installed at each of the 29 plots. Animals which passed an anterior position of each camera were recorded from June 2008 to April 2009, and the data of 480 camera-days during the same period at each plot were used for the consistency across plots.

A total of 3,734 photos of 36 species (36 species in Deramakot and 32 species in Tangkulap) were recorded at the entire plots during the above period (i.e., 13,920 camera-days). Fifteen out of the 36 species are threatened (5 species as “endangered” and 10 species as “vulnerable”) according to the red list of IUCN [13] (Table 1). The mean (±SD) number of species recorded per plot was significantly greater in Deramakot (17.5±2.8; *n* = 20) than in Tangkulap (15.2±2.0; *n* = 9) (P<0.05). Among the 36 species, the following six species showed a significantly higher frequency per plot in Deramakot than in Tangkulap (P<0.05): two ‘vulnerable’ species (Banded palm civet (*Hemigalus derbyanus*) and Sun bear (*Helarctos malayanus*)), one “near threatened” species (Chestnut-necklaced partridge (*Arborophila charltonii*)), three “least concern” species (Borneo yellow muntjac (*Muntiacus atherodes*), Malay civet (*Viverra tangalunga*), and Short-tailed mongoose (*Herpestes brachyurus*)). Greater species diversity and abundance in Deramakot than in Tangkulap are considered to be the result of SFM in the former. In particular, beneficial effects for vertebrates may have been derived from reduced intensity of logging per unit area, more localized logging operations (i.e., restricted to 2–4 compartments per year), and more developed vegetation (as shown in Figure 3).

**Table 1:** Recorded frequency of the 36 vertebrate species per plot in the two forest reserves.

Conservation status	Species	Common names	Deramakot			Tangkulap			p
Endangered	*Pongo pygmaeus*	Orangutan	0.21	±	0.28	0.21	±	0.28	ns
	*Elephas maximus*	Asian elephant	0.11	±	0.29	0.02	±	0.07	ns
	*Manis javanica*	Sunda Pangolin	0.08	±	0.10	0.05	±	0.09	ns
	*Bos javanicus*	Tembadau	0.06	±	0.20	0.05	±	0.14	ns
	*Felis badis*	Bay cat	0.01	±	0.05	0.00	±	0.00	ns
Vulnerable	*Macaca nemestrina*	Pig-tailed macaque	2.36	±	1.67	2.66	±	1.91	ns
	*Sus barbatus*	Bearded pig	2.01	±	1.30	2.75	±	2.32	ns
	*Rusa unicolor*	Sambar deer	0.92	±	0.75	0.86	±	0.65	ns
	*Hemigalus derbyanus*	Banded palm civet	1.25	±	0.90	0.46	±	0.89	*
	*Helarctos malayanus*	Sun bear	0.41	±	0.38	0.16	±	0.20	*
	*Tarsius bancanus*	Western tarsier	0.09	±	0.21	0.25	±	0.27	ns
	*Aonyx cinerea*	Oriental small-clawed otter	0.03	±	0.10	0.12	±	0.18	ns
	*Arctictis binturong*	Binturong	0.03	±	0.08	0.02	±	0.07	ns
	*Neofelis nebulosa*	Clouded leopard	0.03	±	0.08	0.02	±	0.07	ns
	*Paradofelis marmorata*	Marbled cat	0.02	±	0.06	0.02	±	0.07	ns
Near threatened	*Argusianus argus grayi*	Great argus	2.33	±	1.88	1.13	±	1.36	ns
	*Lophura ignita nobilis*	Crested fireback	1.42	±	3.60	0.51	±	0.47	ns
	*Arborophila charltonii*	Chestnut-necklaced partridge	0.34	±	0.52	0.00	±	0.00	**
Least concern	*Trichys fasciculate*	Long-tailed porcupine	2.08	±	2.00	1.94	±	1.68	ns
	*Muntiacus atherodes*	Bornean yellow muntjac	3.20	±	2.31	0.39	±	0.49	**
	*Echinosorex gymnura*	Moonrat	1.34	±	1.34	1.34	±	2.45	ns
	*Viverra tangalunga*	Malay civet	1.58	±	1.83	0.25	±	0.25	**
	*Hystrix brachyura*	Common porcupine	0.61	±	0.95	0.90	±	0.90	ns
	*Mydaus javanensis*	Malay badger	0.67	±	0.73	0.60	±	0.70	ns
	*Paradoxurus hermaphroditus*	Common palm civet	0.29	±	0.55	0.02	±	0.07	ns
	*Herpestes brachyurus*	Short-tailed mongoose	0.24	±	0.19	0.07	±	0.10	**
	*Hystrix crassispinus*	Thick-spined porcupine	0.20	±	0.45	0.09	±	0.15	ns
	*Macaca fascicularis*	Long-tailed Macaque	0.06	±	0.14	0.07	±	0.21	ns
	*Martes flavigula*	Yellow-throated marten	0.08	±	0.14	0.05	±	0.09	ns
	*Prionodon linsang*	Banded linsang	0.06	±	0.17	0.05	±	0.09	ns
	*Prionailurus bengalensis*	Leopard cat	0.03	±	0.08	0.05	±	0.09	ns
	*Arctogalidia trivirgata*	Small-toothed palm civet	0.01	±	0.05	0.00	±	0.00	ns
Least concern & Data deficient	*Tragulus napu* & *Tragulus javanicus*	Greater mouse-deer &Lesser mouse-deer	7.08	±	5.23	5.72	±	1.56	ns
Data deficient	*Herpestes semitorquatus*	Collared mongoose	0.01	±	0.05	0.00	±	0.00	ns
No description	*Varanus salvator*	Water monitor	0.18	±	0.35	0.09	±	0.11	ns
	No of species		17.50	±	2.80	15.22	±	2.05	*

Frequency is given as the number of photographs during 100 days in each plot. The species are grouped according to the IUCN's conservation status [13]. Comparison of means between the two reserves:*, p<0.05; **, p<0.01; ns, not significant.

### Concluding Remarks

Forestry practices have been generally considered destructive and disharmonic with biodiversity conservation. It is true in the sense that they cause negative impacts if applied to pristine forests. However, contemporary landscapes in the tropics and elsewhere are dominated by degraded forests that are legally designated as timber “production forests” [1]. Wildlife including threatened species reside in such degraded forests [14]. Given that strictly protected areas are rather limited in area [15], a pragmatic approach to conserve biodiversity including endangered mammals is to sustainably manage such production forests. SFM with reduced-impact logging applied to degraded natural forests can help to mitigate the deleterious logging impacts on the diversity of vertebrate species as well as the amount of above-ground carbon. If we consider “conventional logging” as a business-as-usual scenario common to many tropical countries, the application of SFM can add carbon and biodiversity in a regional context above such a baseline scenario while maintaining log production. Biodiversity and carbon are the two important ecosystem services of global concern [16], [17], but neither are yet internalized into SFM, because SFM is purely a forestry practice based on timber production. If an international mechanism can be developed to internalize carbon and biodiversity in SFM, SFM will be adopted in much larger areas of natural production forests. If the management approach used in Deramakot were applied to all Bornean production forests, additional 1.88×10^9^ Mg of carbon could be sequestered and a much richer assemblages of forest dwelling vertebrate species could be conserved.

## Methods

### Study Site

This study was carried out in Deramakot (5°14–28′N, 117°19–36′) and Tangkulap (5°17–30′N, 117°11–21′) Forest Reserves in Sabah, Malaysia. The climate of this region is humid equatorial. Its mean annual temperature is 27°C and the mean annual precipitation is about 3,500 mm, with little seasonal variation [7]. Soils in this region are mainly nutrient-poor Acrisols [12]. Altitudes in the reserves range between 20–300 m above sea level, and the entire area is covered with lowland mixed dipterocarp forest.

### Logging History

Several Landsat MSS, TM and ETM+ scenes of the study area were analyzed for describing the disturbance history of the two reserves with different management over a period of 18 years from 1985 until 2002. Taking into account that the land cover type of the study area is lowland mixed dipterocarp forest, the concept of this analysis is based on the fact that reflectance values in the red band can be interpreted as recent disturbances in the crown cover as bare soil is characterized by high reflectance values in the red spectrum, while the undisturbed crown cover of pristine forests shows low reflectance values [18]. High reflectance values of the red band represent two different types of disturbances: 1) Selective logging activities, which occurred within a short period (generally less than one year) prior to the acquisition time of the satellite image. Older logging activities (from former years) cannot be detected due to the fast regrowth of understory. 2) Permanent infrastructures such as major logging roads, which transected both forest reserves and existed during the whole study period. Natural landslides can give rise to a false signal of disturbance, but they are rare in both reserves.

Due to different spatial resolutions of the single Landsat sensors (MSS in comparison to TM and ETM+) all Landsat images were resampled to 80 m pixel size for better inter-sensor comparison. Masks were derived for each of the 6 Landsat scenes (1985, 1991, 1999, 2000, 2001 and 2002), covering cloud and cloud-shadow contaminations. All masks were combined and finally applied to all Landsat imageries in order to retain only those pixels, which showed no cloud or cloud-shadow contamination in any of the 6 scenes. However, when analyzing the time series of Landsat scenes, we experienced the problem that the spectral signal of the red band was influenced by atmospheric effects. Though clouds and cloud shadows were masked, several Landsat scenes were affected by haze, influencing the reflectance values in the red band. However, as haze shows gradual smooth changes over larger areas, it does not influence the relative relationships of the reflectance values among the neighboring pixels and the abruptness in the spatial change of the reflectance among the neighboring pixels is consistent irrespective of haze. In order to separate and finally eliminate these atmospheric influences from real land cover changes on the ground, the slopes of the corresponding red band values were used to estimate the disturbance impact (in Figure 1 referred as ‘Index of disturbance intensity’) instead of directly measuring the reflectance values. The slopes of the reflectance values of the red band of each Landsat imagery were derived using a 3×3 kernel moving window. In Deramakot 12,584 cloud-free pixels were analyzed, while the sample size in Tangkulap was slightly larger with 16,510 sample pixels. Finally, the percent relative area was plotted on the *y*-axis against the disturbance intensity on the *x*-axis double logarithmically. On such a plot, the shape of the curves represents the intensity of the disturbance impact, with convex curves indicating higher disturbance impact than concave curves. Comparison of shape of the curves between Deramakot and Tangkulap was conducted using a Two-sample Kolmogorov-Smirnov test on a 1% significance level.

### Carbon Density

Above- and below-ground carbon density and their spatial variation were estimated using the stratum map of the entire Deramakot and Tangkulap Forest Reserves. The stratum map was originally produced to indicate the spatial variation of stock volume for canopy trees (≥60 cm diameter at breast height (dbh)) using aerial imagery. Panchromatic aerial photographs at a scale of 1∶17,500 taken in 2001 were used for Deramakot, and panchromatic SPOT images of 5 m resolution taken in 2003 were used for Tangkulap. Both materials were visually interpreted to estimate the density of canopy trees based on crown diameter. Although the imagery for Tangkulap was actually taken in 2003, we assumed that the SPOT images reflected the canopy condition of 2001 because the recruitment of canopy trees was negligible during 2001–2003 due to their highly degraded status. The map depicts five classes (i.e., strata) of timber stock: high, mid-high, mid-low, low stratum, and non-forest correspond to densities with ≥16, 9–15, 5–8, 0–4 and 0 trees ≥60 cm dbh per hectare, respectively. The high-stocked forest can be considered as pristine forest, because such a high density of large trees (≥16 trees greater than 60 cm dbh per hectare) appeared only in the forests with minimal levels of anthropogenic disturbance in this region (Imai et al. unpublished).

We established two to four 0.2 ha rectangular [19] or 0.12 ha circular plots (J. Titin unpublished) in each stratum, as well as one 2 ha plot in each of the mid-high, mid-low, and low density strata during 2005–2008. All trees ≥10 cm dbh were measured in each plot. Aboveground biomass was estimated according to Brown's allometric equation [20].

In two out of three large plots, we collected wood samples of canopy dominant species, which were defined as species with ≥3% of the relative basal area to estimate aboveground carbon stocks. Three trees were sampled from each of the canopy dominant species. Wood samples were extracted from the outer sapwood area 1 m from the ground using an increment borer. We collected at least two wood samples per individual tree, and combined the samples by individual tree.

To estimate the stock of soil organic carbon in the upper 1 m, we excavated triplicate soil pits in each of three topographic positions (flat ridge (1.8–10°), gentle (10–30°) and steep slopes (30–43.7°)) in each of the three 2 ha plots (i.e., 9 soil pits per plot). The O horizon comprising fresh litter and humus was sampled at three random points around each pit using a circular frame (23 cm diameter). At the same sampling point below the O horizon, A horizon (0–5 cm deep) was sampled using a 5 cm-deep core sampler. Deeper samples were collected from the walls of soil pits using the same sampler. In nine out of the 11 small plots, we set four 40 m line transects per plot, sampled O and A horizons by the same methods at 10 m intervals on each line transect, combined samples of each transect, and made four composite samples for each plot. We sampled only O and A horizons in small plots, because carbon storage at AB and B horizon did not significantly differ between the three 2 ha plots. Samples of A, AB and B horizons were sorted into soils and living fine roots <2 mm diameter. O horizon was sorted into living fine roots, leaves and twigs <2 cm girth. Our estimation of carbon density does not include coarse roots (≥2 mm diameter), coarse woody debris (≥2 cm girth), trees <10 cm dbh, herbs and lianas.

All vegetative and soil samples were dried, weighed, finely ground, and analyzed for carbon concentration by the dry combustion method with an N-C analyzer (JM1000CN, J-Science Lab, Kyoto). Total carbon density, the sum of above-vegetation, fine roots, and soil organic carbon on an area basis was obtained by multiplying the mass of each component by its corresponding weight-basis concentration in each plot. Subsequently, the mean (±SD) of the replicated plots in each stratum was determined. We weighed the mean (±SD) of the above-, below-, and total-ecosystem carbon density of each stratum with its relative land area in each reserve to determine the mean (±SD) of the entire reserve. Significant difference in the mean carbon density of above- and below-ground system among the four strata was tested by an analysis of variance (ANOVA), followed by the Tukey-Kramer *post-hoc* test at *p* = 0.05. In this analysis, we omitted the non-forest stratum because it covered <2% of each area.

We estimated the amount of additionally sequestered carbon if sustainable forest management (SFM) were applied to all production forests in entire Borneo. In this analysis, we assumed that all production forests were as degraded as Tangkulap. The mean difference of carbon density between Tangkulap and Deramakot (54 t C ha^−1^, see the text) was then multiplied by the area of production forests in Borneo (i.e. 35,000,000 ha; K. Kitayama unpublished). This estimate of the difference in carbon density between Tangkulap and Deramakot is conservative because the majority of the contemporary landscape may be much more degraded than Tangkulap.

### Frequency and Diversity of Medium to Large-Bodied Forest-Dwelling Vertebrates

We estimated the distribution and diversity of medium to large-bodied forest-dwelling vertebrates throughout Deramakot and Tangkulap. In June 2008, we systematically selected 29 circular plots each with 500 m radius at approximately 5 km intervals in the two forest reserves. At each plot, three passive heat-sensor cameras (Filed Note II, Marifu, Iwakuni, Japan) were placed along the animal track closest to a randomly selected point (generally <20 m). A camera was attached to an appropriate standing tree at about 1 m height and automatically photographed all animals passing at an anterior position of the camera in all hours. Films and batteries were replaced monthly, and the location of the three cameras was shifted to other random points every three to four months for a total of three times per plot, i.e. nine camera points per plot. We conducted the census from June 2008 to April 2009 and obtained data of 480 camera-days per circular plot despite some mechanical failures of some cameras. The frequency with which each species was photographed was calculated for each plot. Differences in the mean number of species and observed frequency of each species per plot between Deramakot and Tangkulap were tested by Welch Two Sample t-test. We counted only forest-dwelling vertebrate species >1 kg weight, which included 32 mammals, three terrestrial birds, and one reptile. There is no local settlement within the two reserves and hunting activity is prohibited and strictly monitored by Forestry Department. Therefore, hunting pressure is minimal in both reserves.
